# Methylglyoxal—A Potential Risk Factor of Manuka Honey in Healing of Diabetic Ulcers

**DOI:** 10.1093/ecam/neq013

**Published:** 2010-10-14

**Authors:** Juraj Majtan

**Affiliations:** ^1^Institute of Zoology, Slovak Academy of Sciences, 845 06 Bratislava, Slovakia; ^2^Department of Microbiology, Slovak Medical University, Bratislava, Slovakia

## Abstract

Honey has been considered as a remedy in wound healing since ancient times. However, as yet, there are inadequate supportive robust randomized trials and experimental data to fully accept honey as an effective medical product in wound care. Manuka honey has been claimed to have therapeutic advantages over other honeys. Recently, it has been documented that the pronounced antibacterial activity of manuka honey is due, at least in part, to reactive methylglyoxal (MG). The concentration of MG in manuka honeys is up to 100-fold higher than in conventional honeys. MG is a potent protein-glycating agent and an important precursor of advanced glycation end products (AGEs). MG and AGEs play a role in the pathogenesis of impaired diabetic wound healing and can modify the structure and function of target molecules. This commentary describes the concern that MG in manuka honey may delay wound healing in diabetic patients. Further detailed research is needed to fully elucidate the participation of honey/derived MG in healing diabetic ulcers. We advocate randomized controlled trials to determine efficacy and safety of manuka honey in this population.

## 1. Introduction

The prevalence of diabetes is rapidly increasing worldwide. The occurrence of diabetic foot ulcers is a serious complication which negatively affects patients' quality of life. Among persons diagnosed with diabetes mellitus, the prevalence of foot ulcers is 4%–10% [1]. Treatment of foot ulcers is protracted and intensive and associated with high costs. Various approaches have been developed for the treatment of diabetic ulcers including topical wound-care therapies.

Current therapeutic products which are being used widely in wound care, such as silver sulfadiazine, hydrogel, hydrocolloid and alginate dressings impregnated with silver, are generally accepted as being useful for control of bacterial infections. Increased use of ionic silver in wound care has created some concern regarding the development of bacterial resistance [2, 3]. Silver resistance has already been documented in bacterial isolates including *Escherichia coli* [4], *Enterobacter cloacae* [4], *Klebsiella pneumonie* [4], *Salmonella typhimurium* [5] and *Acinetobacter baumani* [6].

With increasing frequency, modern medicine directs attention to natural products with antimicrobial activity and their use in clinical practice. The major arguments for implementing natural products such as honey, aloe vera or curcumin are the low cost and absence of antimicrobial resistance risk in comparison with conventional wound-care products. Moreover, some of the natural therapies such as maggot debridement therapy and phage therapy have recently been reexamined for their potential usefulness in the treatment of wounds that are difficult to heal [7–10].

## 2. Honey as a Traditional Antimicrobial Agent

**Figure 1:**
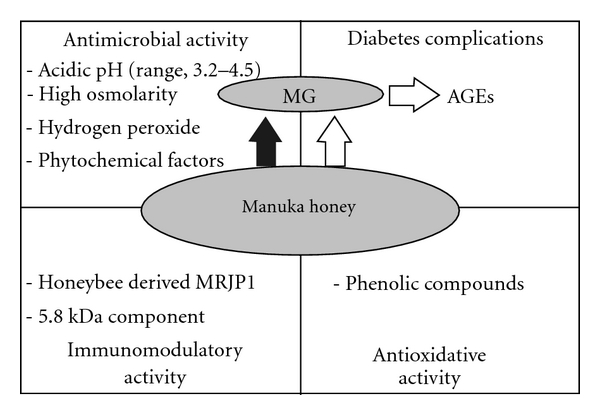
Main properties of manuka honey in the treatment of diabetic ulcers. The black arrow represents known action, the white arrows represent hypothetical mechanisms of action. MG: methylglyoxal; AGEs: advanced glycation end products; MRJP1: major royal jelly protein 1.

Historically, honey has been used in the treatment of a broad spectrum of wounds. It is well documented that honey can act as an antimicrobial and antioxidative agent as well as an immunomodulator with both pro- and anti-inflammatory effects [11–15] (Figure 1). Recent research has concentrated on the antimicrobial activity of different types of honey rather than its effect on wound healing [16–20]. Honey derived from the floral source of *Leptospermum* spp. (manuka) has been claimed to have therapeutic advantages over other honeys [21–23]. Manuka honeys exhibit notable antimicrobial activity which cannot be attributed to the peroxide present [24, 25]. It has been documented that the pronounced antibacterial activity of manuka honey directly originates from the methylglyoxal (MG) it contains [26, 27]. Honey derived from this specific plant in New Zealand and Australia gave rise to Medihoney—widely used medical honey. George and Cutting [28] compared the sensitivity of 130 clinical isolates to this medical honey. They demonstrated that manuka honey is effective against a broad range of microorganisms including multi-resistant strains [28]. In a very recent study it has been reported that manuka honey has antimicrobial activity against clinical isolates of *Campylobacter* spp. [29]. Along with MG, the hydrogen peroxide, flavonoids as well as aromatic acids present in natural honeys also act as antimicrobial substances.

## 3. Clinical Evidence for the Use of Honey in the Management of Chronic Wounds

The findings of animal studies and of several randomized clinical trials involving more than 2000 participants have provided compelling evidence that honey can accelerate wound healing [22]. On the other hand, the quality of reported trials is variable and evidence to date supports honey only as a treatment of mild-to-moderate superficial and partial thickness burns [30]. Authors of a recent systematic review assert that there is insufficient evidence to guide clinical practice for other wound types [30]. Jull and co-workers established that honey-impregnated dressings did not significantly improve venous ulcer healing at 12 weeks compared with usual care [31]. Moreover, these dressings did not significantly improve time to healing, change in ulcer area, incidence of infection or quality of life. On the other hand, two very recent clinical trials suggest that healing times and incidence of infection after treatment with honey are reduced compared to conventional treatment, and the results are of clinical significance [32, 33]. The main limitation of above two trials is that patient numbers recruited for inclusion were insufficient to reach statistical significance.

Manuka honey is the type of honey most often studied in randomized controlled studies. Six trials (*n* = 701) [31–36] recruited participants with chronic wounds, three of which recruited participants with leg ulcers [31–33]. In all three trials manuka honey dressing was used. The participants with history of diabetes were excluded from two trials [31, 33]. Although, diabetic participants with venous ulcers were not excluded in the remaining trial [32], the effect of manuka honey in the treatment of ulcers in patients with diabetes was not specifically examined.

Non-manuka honey was recently compared to povidone iodine in the treatment of Wagner type II diabetic foot ulcers [37]. Ulcer healing was not significantly different in both groups and the authors deduced that honey dressing represents a safe alternative dressing for treatment of diabetic foot ulcers. In addition, Eddy and Gideonsen [38] reported a case where ordinary honey was applied to patient's heel and forefoot diabetic ulcers in order to avoid the leg amputation. Granulation tissue appeared within 2 weeks and the ulcers healed in 6–12 months.

The effect of different honey on healing has not been compared *in vivo* in either the animal or the human model. It would be interesting to compare effectiveness of several types of honey in randomized controlled clinical trials recruiting participants with diabetic ulcers. It has been described that the Slovak forest honey has comparable antibacterial activity to manuka honey against nosocomial and skin pathogens *in vitro* [20].

## 4. Methylglyoxal Concentrations in Honey

MG and other dicarbonyls have been identified in a range of foods and beverages, including wine [39], beer [39], bread [40], soya [41] and honey [42]. MG in manuka honeys is derived from the non-enzymatic conversion of dihydroxyacetone which occurs at high levels in the nectar [42]. Freshly produced manuka honey contained low levels of MG (139–491 mg kg^−1^) but during storage at 37°C its content increased. The levels of MG in multi-floral honeys are low, ranging from 0.4 to 5.4 mg kg^−1^ [43]. In two studies, high levels of MG were reported in manuka honeys ranging from 48 to 743 mg kg^−1^ [27] and 189 to 835 mg kg^−1^ [44], respectively. It has been suggested that concentrations of MG above 150 mg kg^−1^ are directly responsible for the characteristic antibacterial properties of manuka honey [44].

## 5. Biological Properties of MG and AGEs

The 1,2-dicarbonyl compounds, including MG, glyoxal and 3-deoxyglucosone, are generated either endogenously by cell metabolism, glucose oxidation and lipid peroxidation or by degradation of carbohydrates in foods and beverages. Highly reactive dicarbonyls attack the lysine, arginine (Arg) and cysteine residues of long-lived proteins, such as collagens, to form irreversible AGEs causing changes in collagen pathophysiology that result in disruption of normal collagen matrix remodeling [45].

AGEs are complex, heterogenous molecules that cause protein cross-linking, exhibit browning and generate fluorescence [46]. Not all AGEs have been identified and the mechanisms underlying their formation remain unclear. AGEs exert their actions either directly or through a receptor system that includes two types of cell-surface AGE receptors [47, 48]. It has been found that a large portion of AGEs and their precursors in the body are derived from exogenous sources [49, 50].

MG-induced modifications of Arg residues in collagen could be a key factor in the impaired degradation of collagen that promotes fibrosis in chronic tissue infections [51]. Interestingly, MG increased the apparent molecular weight of collagen, which is suggestive of increased collagen cross-linking [45]. MG also changes the structure and function of many important immunological proteins and enzymes, and reduces the efficiency of peripheral blood immune-cell response [52]. The administration of MG to rats induced severe negative changes in cutaneous vessels, suggesting decreased peripheral perfusion by an impaired microcirculation [53]. Furthermore, authors observed that ulcers in MG-treated rats failed to provide actively migrating cells from peripheral regions.

Treatment of early-passage human skin fibroblasts with 400 *μ*M of MG results in accelerated aging and the appearance of the senescent phenotype [54].

## 6. The Roles of MG and AGEs in Impairment of Diabetic Wound Healing

The scope of MG damage in diabetes is huge, because MG is able to form adducts on protein, lipoproteins and DNAs at any site where its concentration is high. It has also been suggested that MG glycation could be a link between diabetes and infection susceptibility [52]. MG-induced immune damage might provide a link between hyperglycemia and diabetes-related infection risk [52].

Data from recent experimental studies indicate that not only MG but also AGEs may play a role in the pathogenesis of impaired diabetic wound healing and might have separate consequences for immunity [54–58]. Increased AGE accumulation, along with expression of their receptors, are associated with formation of diabetic atheromatous lesions, triggering their destabilization, impairing ischemia-induced neovascularization and formation of collateral circulation, expanding vessel extracellular matrix, and disrupting the expression of endothelial nitrous oxide synthetase [47, 48, 58, 59].

The accumulation of AGEs in the extracellular matrix causes aberrant cross-linking, resulting in decreased vessel elasticity. It has also been shown that keratinocyte migration is inhibited by AGEs accumulation under high-glucose conditions [60]. In diabetes, AGEs can enhance the activity of neutrophils, with change in cellular biological behaviors which might be one of the main reasons responsible for chronic inflammation [61]. The sustained inflammatory response occurs following interaction of AGEs with their receptors and release of pro-inflammatory mediators such as tumor necrosis factor *α* and production of destructive matrix metalloproteinases which limit wound closure [56]. In addition, Goova et al. [56] have demonstrated that blocking receptors for AGEs restores impaired wound healing in diabetic mice.

## 7. Conclusion

A number of randomized clinical trials suggest that medical grade honey promotes wound healing but most of them have not included diabetic patients with chronic leg ulcers and nearly three-quarters of clinical trials have focused on the use of honey for acute wounds, in particular burns. In general, repair of tissue damage in diabetic foot disease is accompanied with several pathophysiological mechanisms. Since manuka honey contains high levels of MG, we speculate that patients with diabetes may be at risk due to either the direct negative effect of MG on cells and components in the wound or the indirect formation of AGEs, which could impair the wound-healing process.

We believe that honey is an effective alternative dressing for treatment of many types of chronic wounds. However, MG may have a detrimental effect on diabetic ulcers. Further research is needed to fully elucidate the effect of honey-derived MG in the treatment of diabetic ulcers. In addition, honeys with varying levels of MG should be subjected to randomized comparative clinical trials in treatment of diabetic ulcers.
